# Anti-Inflammatory Effect of Recreational Exercise in TNBS-Induced Colitis in Rats: Role of NOS/HO/MPO System

**DOI:** 10.1155/2014/925981

**Published:** 2014-02-06

**Authors:** Zita Szalai, András Szász, István Nagy, László G. Puskás, Krisztina Kupai, Adél Király, Anikó Magyariné Berkó, Anikó Pósa, Gerda Strifler, Zoltán Baráth, Lajos I. Nagy, Renáta Szabó, Imre Pávó, Zsolt Murlasits, Mariann Gyöngyösi, Csaba Varga

**Affiliations:** ^1^Department of Physiology, Anatomy and Neuroscience, University of Szeged, Közép Fasor 52, Szeged 6726, Hungary; ^2^Institute of Physical Education and Sport Sciences, University of Szeged, Szeged 6725, Hungary; ^3^Institute of Biochemistry, Biological Research Centre of the Hungarian Academy of Sciences, Szeged 6726, Hungary; ^4^AVIDIN Ltd., Szeged 6726, Hungary; ^5^Institute of Genetics, Biological Research Center of the Hungarian Academy of Sciences, Szeged 6726, Hungary; ^6^Semmelweis University, Faculty of Physical Education and Sport Sciences, Budapest 1123, Hungary; ^7^Department of Cardiology, Medical University of Vienna, 1090 Wien, Austria; ^8^University of Szeged, Faculty of Dentistry and Department of Orthodontics and Pediatric Dentistry, Szeged 6720, Hungary

## Abstract

There are opposite views in the available literature: Whether physical exercise has a protective effect or not on the onset of inflammatory bowel disease (IBD). Therefore, we investigated the effects of recreational physical exercise before the induction of colitis. After 6 weeks of voluntary physical activity (running wheel), male Wistar rats were treated with TNBS (10 mg). 72 hrs after trinitrobenzene sulphonic acid (TNBS) challenge we measured colonic gene (TNF-**α**, IL-1**β**, CXCL1 and IL-10) and protein (TNF-**α**) expressions of various inflammatory mediators and enzyme activities of heme oxygenase (HO), nitric oxide synthase (NOS), and myeloperoxidase (MPO) enzymes. Wheel running significantly increased the activities of HO, constitutive NOS (cNOS) isoform. Furthermore, 6 weeks of running significantly decreased TNBS-induced inflammatory markers, including extent of lesions, severity of mucosal damage, and gene expression of IL-1**β**, CXCL1, and MPO activity, while IL-10 gene expression and cNOS activity were increased. iNOS activity decreased and the activity of HO enzyme increased, but not significantly, compared to the sedentary TNBS-treated group. In conclusion, recreational physical exercise can play an anti-inflammatory role by downregulating the gene expression of proinflammatory mediators, inducing anti-inflammatory mediators, and modulating the activities of HO and NOS enzymes in a rat model of colitis.

## 1. Introduction

Inflammatory bowel disease (IBD) is a chronic disease of the gastrointestinal tract, which primarily comprises Crohn's disease (CD) and ulcerative colitis. Physical exercise can be either harmful or beneficial for the gastrointestinal (GI) tract depending on the training variables, such as exercise intensity. For instance, it is believed that the reduced blood flow to the gut during exercise can disturb the GI system [1]. Although there are similar studies suggesting the preventive effect of active lifestyle on the onset of IBD, the available literature is weak and contradictory [2, 3].

In the healthy gut interleukin-10 (IL-10) is the key cytokine responsible for the anti-inflammatory environment, primarily through the suppression of proinflammatory cytokines [4]. Once the anti- and proinflammatory balance of the intestinal immune system is disturbed, the activated and stimulated macrophages and T lymphocytes release proinflammatory mediators such as tumour necrosis factor-*α* (TNF-*α*), interleukin-1*β* (IL-1*β*), and chemokine ligand 1 (CXCL1). The importance of anti- and proinflammatory balance is reflected in the efficacy of anti-TNF-*α* therapy in the treatment of IBD [5]: TNF-*α* inhibitors have proven successful in inducing and maintaining remission of moderate-to-severe IBD. In addition, CXCL1 is a chemoattractant for neutrophils and plays a role in inflammation; increased ileal and colonic CXCL1 gene expression was also reported in IBD patients [6].

IBD is associated with enhanced production of reactive metabolites of oxygen and nitrogen (RONS). Moreover, oxidative stress plays an important role in the pathogenesis of this disease [7]. Heme oxygenase-1 (HO-1), the inducible isoform of heme oxygenase enzymes, is a stress-responsive enzyme, which can be induced by oxidative stress, inflammatory cytokines, and many other molecules. HO-1 is thought to play an important role in the protection of tissues from oxidative injuries and inflammation [8–10]. These beneficial effects of HO-1 are mediated in part by the antioxidant and anti-inflammatory end products of heme breakdown, namely, CO and bilirubin, which can inhibit the expression of proinflammatory cytokines, such as TNF-*α* and IL-1*β*. Several molecules, such as interleukin-10 (IL-10) function through an HO-1 dependent mechanism [11, 12]. Pharmacological studies suggest a beneficial role for HO-1 in modulating colitis. Upregulation of HO-1 by heme and cadmium chloride can ameliorate while inhibition of the enzyme by tin protoporphyrin can aggravate experimental inflammation in the colon [13]. 5-Aminosalicylic-acid, a clinically approved anticolitic agent, can exert beneficial effects in vivo, partially through the induction or activation of HO-1 [14].

Another enzyme involved in intestinal inflammation and oxidative stress is nitric oxide synthase [15]. The constitutive production of NO derived from the constitutive isoforms of the enzyme, namely, neuronal NOS (nNOS) and endothelial NOS (eNOS), plays a role in various physiological processes in the GI mucosa, such as the regulation of microvascular and epithelial permeability and the maintenance of adequate perfusion [16]. On the other hand the effects of iNOS-produced [15] NO can be beneficial or detrimental depending on the amount, duration, and anatomical site of synthesis [16, 17]. In experimental colitis the downregulation of nNOS and the overproduction of NO by iNOS are observed [18]. For example, proinflammatory cytokines, such as TNF-*α*, can induce NO production and iNOS activity in colonic epithelial cells [17].

Earlier studies have focused mainly on the role of exercise in the treatment of extraintestinal manifestations of IBD [19, 20]. However, the effects of physical exercise on the inflamed gut are still inconsistent. Therefore, the aims of the present study were to investigate the effects of recreational physical exercise (1) on the colonic damage in a TNBS rat model (damage score, lesion), (2) on the gene expression (TNF-*α*, IL-1*β*, CXCL1, and IL-10) and protein concentration (TNF-*α*) of inflammatory mediators in experimental acute colitis, and (3) on the activity of oxidative/antioxidative enzymes (MPO, HO, and NOS) in the inflamed colon of rats. We hypothesized that voluntary exercise prior to TNBS challenge will reduce colonic damage and oxidative and inflammatory mediator gene and protein expressions.

## 2. Materials and Methods

### 2.1. Animals

Male Wistar rats (180–220 g) were housed in groups. Food was withdrawn overnight before induction of colitis; otherwise, the animals had access to food and drinking water ad libitum throughout the experiments. The animal care and research protocols were in accordance with the guidelines of the University of Szeged.

### 2.2. Experimental Design

**Figure 1:**
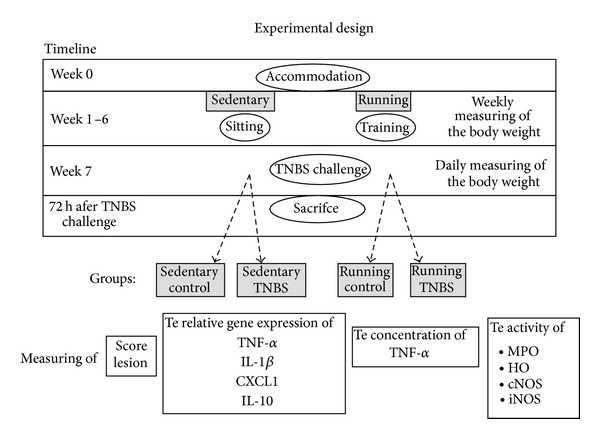
Diagram showing the experimental design.

The animals were randomly divided into four groups: 1: sedentary control (nonrunning non-colitis-induced, *n* = 13), 2: running control (running non-colitis-induced, *n* = 18), 3: sedentary TNBS (*n* = 14), and 4: running TNBS groups (*n* = 18) (Figure 1). Rats of the running groups were placed in cages with a running wheel (Acellabor Ltd., Budapest, Hungary). Before the beginning of experimental period rats were allowed to accommodate to the running wheel for a week. The activity of the rats on the running wheel was monitored by a bicycle computer attached to each wheel. After 6 weeks of self-administered physical exercise colitis was induced by 2,4,6-trinitrobenzene sulphonic acid (TNBS; once 10 mg in 0.25 mL of 50% ethanol, w/v). The intracolonic administration of TNBS was performed with an 8 cm long plastic catheter under transient ether anaesthesia (the method originally described by Morris et al. [21]). The control groups did not receive any treatment. Physical activity was continued following TNBS treatment. Rats were weighed twice a week until the induction of colitis and then daily following the TNBS challenge. 72 hours after the induction of colitis the animals were sacrificed and the distal 8 cm portion of the colon was dissected, longitudinally opened, gently rinsed with ice-cold physiological saline, and photographed (Panasonic Lumix DMC-TZ6, digital camera) for the determination of macroscopic colonic inflammatory damage. The colon was weighed and divided into longitudinal segments to be used for the following molecular and biochemical analyses.

### 2.3. Damage Score and Lesion Measurement

The extent of macroscopically apparent inflammation, ulceration, and tissue disruption was determined in a randomized manner from the colour images, using a proprietary computerized planimetry software, developed in our laboratory (Stat_2_1_1). The area of macroscopically visible mucosal involvement was calculated and expressed as the percentage of the total colonic segment area under study.

The degree of colonic inflammation was scored on a 0–11 scale in a randomized, blinded fashion. The criteria has been adapted from what has been used previously: 0 = no damage, 1 = focal hyperemia and no ulcers, 2 = ulceration without hyperemia or bowel wall thickening, 3 = ulceration with inflammation at 1 site, 4 = more than 2 sites of ulceration and inflammation, 5 = more than 2 major sites of ulceration and inflammation or 1 site of ulceration/inflammation extending >1 cm along the length of the colon and 6–11 = the score is increased by 1 for each additional centimetre of involvement [14].

### 2.4. Quantification of TNF-*α* Protein with the AlphaLISA Assay

The rat large intestine was immediately frozen in liquid nitrogen after dissection. During protein extraction the tissues were stored on ice. We used 1 mL RIPA buffer (150 mM NaCl (Molar Chemicals, Budapest, Hungary), 1% Nonidet P-40 (Sigma-Aldrich), 0.5% Na-deoxycholate (Sigma-Aldrich), 0.1% sodium dodecyl sulfate (Sigma-Aldrich), and 50 mM Tris-HCl (pH 8.0) (Molar Chemicals)) for protein extraction from 40 mg tissue, supplemented with 20 *μ*L NEM (N-ethyl-maleinimide) and 20 *μ*L phenyl-methane-sulfofluoride (PMSF) protease inhibitors. We homogenized the tissue samples with Ultra-Turrax T25 (IKA-Labortechnik, Staufen, Germany) tissue homogenizer on ice for 1 min. The homogenates were centrifuged for 10 min at 10.000 ×g at 4°C and the concentration of the supernatant was measured with a NanoDrop ND-1000 spectrophotometer (NanoDrop Technologies, Wilmington, USA). All samples were then diluted to a uniform 0.5 mg/mL concentration. Until further analyses samples were stored at −20°C (stock samples were stored at −80°C).

For the quantification of TNF-*α*, we used the AlphaLISA bead based technique, which was developed and is supported by Perkin Elmer (Perkin Elmer, Massachusetts, USA). Alpha Technology is a highly sensitive assay ideal for the measurement of protein interaction. We used 5 *μ*L volume from 0.5 mg/mL protein solution. The final amount of AlphaLISA Anti-Analyte Acceptor beads was set at 10 *μ*g/mL concentration, while the final amount of Streptavidin-Donor beads was set at 40 *μ*g/mL. The final working concentration of Biotinylated Antibody Anti-Analyte was 1 nM. All incubations were under subdued laboratory lighting (<100 lux). The samples were measured in 50 *μ*L final volume filled in 384-well polystyrene plates (Tomtec Plastik, Budapest, Hungary) with an EnVision-Alpha Reader (PerkinElmer).

### 2.5. Myeloperoxidase Activity

To measure myeloperoxidase activity we employed a modification of the method described by Bradley et al. [22]. The 8 cm longitudinal strips of the colon were weighed, homogenized (Ultra-Turrax T25 IKA-Labortechnik, 13.500 rev/min, twice for 30 s; 250 mg colon/mL buffer) in ice-cold phosphate buffer (50 mM, pH 6.0), containing 0.5% hexadecyltrimethylammonium-bromide, freeze-thawed three times, and then centrifuged at 10.000 ×g for 15 min at 4°C. A 12 *μ*L aliquot of the supernatant was then mixed with 280 *μ*L phosphate buffer (50 mM, pH 6.0), containing 0.167 mg/mL O-adenosine dihydrochloride (Sigma-Aldrich), and the reaction was started with 10 *μ*L 0.03% hydrogen peroxide and assayed spectrophotometrically at 490 nm after 90 sec shaking (Benchmark Microplate Reader, Bio-Rad Labs, Hercules, CA). MPO activity was expressed as mU/mg protein.

### 2.6. Heme Oxygenase Activity

Heme oxygenase activity was assessed by measuring bilirubin formation with slight modifications form what has been described by Tenhunen et al. [23]. The segment of the colon was homogenized (Ultra-Turrax T25; 13.500/s; twice for 30 sec) in ice-cold 10 mM N-[2-hydroxyethyl] piperazine-N′-[2-ethanesulfonic acid] (HEPES, Sigma-Aldrich), 32 mM sucrose (Sigma-Aldrich), 1 mM dithiothreitol (DTT, Sigma-Aldrich), 0.1 mM EDTA, 10 *μ*g/mL soybean trypsin inhibitor (Sigma-Aldrich), 10 *μ*g/mL leupeptin (Sigma-Aldrich), and 2 *μ*g/mL aprotinin (Sigma-Aldrich), at pH 7.4. The supernatant was collected by centrifugation for 30 min at 20.000 ×g at 4°C. Incubation was carried out in the dark at 37°C for 60 min with the reaction mixture containing the following ingredients in a final volume of 1.5 mL : 2 mM glucose 6-phosphate (Sigma-Aldrich), 0.14 U/mL glucose 6-phosphate dehydrogenase (Sigma-Aldrich), 15 *μ*M heme, 150 *μ*M *β*-nicotinamide adenine dinucleotide phosphate (*β*-NADPH, Sigma-Aldrich), 120 *μ*g/mL rat liver cytosol as a source of biliverdin reductase, 2 mM MgCl_2_, 100 mM potassium phosphate buffer, and 150 *μ*L of supernatant. The reaction was stopped by placing the samples on ice. The bilirubin formed was calculated from the difference between optical densities obtained at 460 and 530 nm. One unit of heme oxygenase activity was defined as the amount of bilirubin (nmol) produced/hour/mg protein.

### 2.7. Nitric Oxide Synthase Activity

Nitric oxide synthase activity was determined by quantifying the conversion of [^14^C]-radiolabelled L-arginine to citrulline by a previously described method with some minor modifications [24]. A segment of colon was homogenized as described for HO activity. Homogenates were centrifuged for 30 min at 20.000 ×g at 4°C. Samples (40 *μ*L) were incubated for 10 min at 37°C in 100 *μ*L of assay buffer (50 mM KH_2_PO_4_, 1.0 mM MgCl_2_, 50 mM L-valine, 0.2 mM CaCl_2_, 1.0 mM DTT, 1.0 mM L-citrulline, 15.5 nM L-arginine, 30 *μ*M flavin adenine dinucleotide, 30 *μ*M flavin mononucleotide, 30 *μ*M tetrahydro-L-biopterin dihydrochloride, 450 *μ*M *β*-NADPH, and 12 pM of [^14^C]-L-arginine monohydrochloride (all from Sigma-Aldrich)). The reaction was terminated by the addition of 0.5 mL of 1 : 1 (v/v) suspension of ice-cold DOWEX (Na^+^-form) in distilled water. The mixture was resuspended with the addition of 850 *μ*L of ice-cold distilled water. The supernatant (970 *μ*L) was removed and radioactivity was determined by scintillation counting. Calcium-dependency of the NOS activity was determined by the addition of 10 *μ*L of ethylene glycol-bis (*β*-aminoethyl ether) tetraacetic acid (EGTA; 1 mM, Sigma-Aldrich). NOS activity was confirmed by inhibition with 10 *μ*L of N**ω**-nitro-L-arginine-methylester (LNNA; 3.7 mM, Sigma-Aldrich). Inducible NOS was defined as the citrulline formation that was inhibited by LNNA, but not by EGTA. The constitutive NOS activity was calculated from the difference between citrulline formation that was inhibited by EGTA and the total activity. As the nature of the constitutive isoform (eNOS or nNOS) was not determined, this activity is referred to as cNOS. NOS activity was expressed as pmol/min/mg protein.

### 2.8. Protein Determination for HO, NOS, and MPO Activity

Using a commercial protein assay kit (Bio-Rad Labs), aliquots (20 *μ*L) of the diluted samples (15× or 25× with distilled water) were mixed with 980 *μ*L of distilled water with 200 *μ*L Bradford reagent added to each sample. After mixing and following 10 min incubation, the samples were assayed spectrophotometrically at 595 nm. Protein level was expressed as mg protein/mL.

### 2.9. RNA Extraction, Reverse Transcription, and Quantitative Reverse Transcriptase Polymerase Chain Reaction (QPCR)

The samples were homogenized in 1 mL of TRIzol reagent (Life Technologies, Carlsbad, CA) with ultra-turrax T-18 basic homogenizer (2 × 30 sec at 5000 rpm); one-third volume of chloroform (Sigma-Aldrich) was added to the suspension with vigorous vortexing. Samples were centrifuged at 13000 rpm for 10 minutes and total RNA was extracted from the upper phase by using RNeasy Plus Mini Kits (Qiagen, Germany) according to the manufacturer's protocol. In parallel, peripheral blood mononuclear cells (PBMC) were isolated from the blood of the same animals by Ficoll Paque Plus (GE Healthcare, UK) density gradient centrifugation. Total RNA from PBMC was also extracted using RNeasy Plus Mini Kits (Qiagen). The quality and quantity of isolated RNA were determined using NanoDrop and Bioanalyzer (Agilent, Santa Clara, USA) measurements.

cDNA was synthesized from 100 ng of total RNA by using High Capacity RNA to cDNA Kit or SuperScript Vilo cDNA Synthesis Kit (both from Life Technologies) according to the manufacturer's instructions. cDNA levels were determined by QPCR using StepOne Plus Real-Time PCR System (Life Technologies). Reactions were performed with Power SybrGreen Master Mix (Life Technologies) with the following primer sets:

 TNF-*α* sense: GCTCCCTCTCATCAGTTCCA,
 TNF-*α* antisense: GGCTTGTCACTCGAGTTTTGA;
 CXCL1 sense: CATTAATATTTAACGATGTGGATGCGTTTCA,
 CXCL1 antisense: GCCTACCATCTTTAAACTGCACAAT [25];
 IL-1*β* sense: CAGGAAGGCAGTGTCACTCA,
 IL-1*β* antisense: AGACAGCACGAGGCATTTTT;
 IL-10 sense: CCTGCTCCTACTGGCTGGAG,
 IL-10 antisense: TTGTTCAGCTGGTCCTTCTT.

To avoid false-positive results due to amplification of contaminating genomic DNA in the cDNA preparation, we used primers spanning exon-exon junctions. All measurements were performed in duplicates with at least four biological replicates. The ratio of each mRNA relative to the 18S rRNA (assay ID: Hs99999901; Life Technologies) was calculated using the ΔΔ*C*_*T*_ method.

### 2.10. Data Representation and Statistical Analysis

Results of the body weight change, damage score, lesion, and the activity of MPO, HO, cNOS, and iNOS are shown as mean ± S.E.M; statistical comparisons were performed by two-tailed Student's *t*-test. Statistical analysis of the TNF-*α* protein secretion was performed using one-way ANOVA. QPCR data are presented as data points and median; the significance of the differences between samples was determined by applying the Newman-Keuls test using GraphPad Prism for Windows. In all statistical comparisons, a probability (*P*) value of less than 0.05 was considered significant.

## 3. Results

### 3.1. Alteration of Body Weight after Running and TNBS Treatment

**Figure 2:**
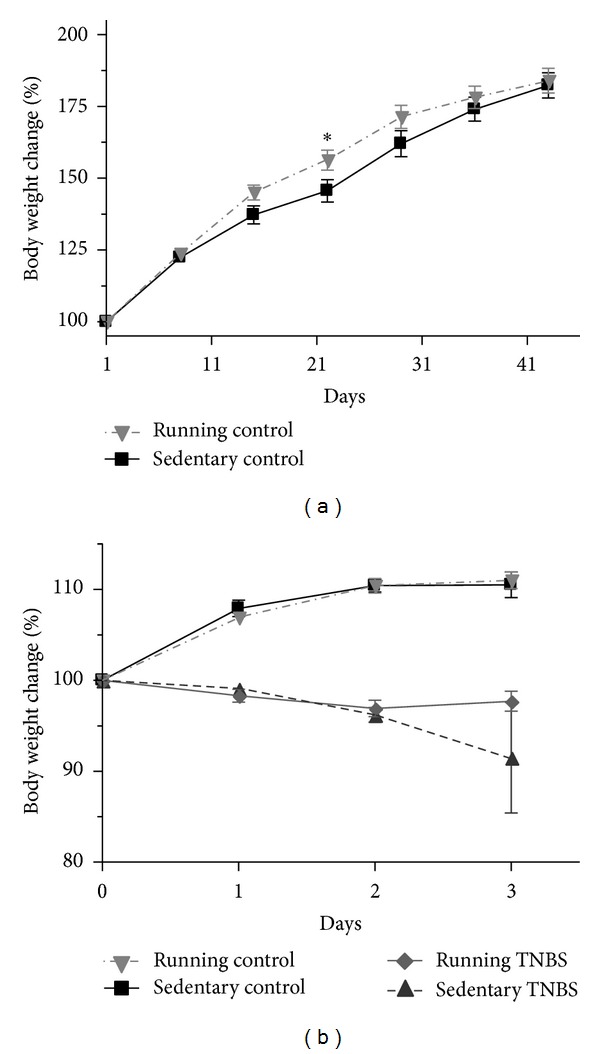
Body weight changes during the 6 weeks of running (a) and after the TNBS treatment (b). Results are shown as mean ± S.E.M. (*P < 0.05 compared to the sedentary nontreated control group, *n* = 34–43).

Running distance averaged 2600 meters/day/rat. We did not find any differences between the body weights of sedentary and running groups during the 6 weeks of exercise before TNBS administration (Figure 2(a)). On the other hand, there was a progressive decrease in body weight after the induction of colonic inflammation in the TNBS treated groups (Figure 2(b)).

### 3.2. Effects of 6-Week Running and TNBS Challenge on Damage Score, Mucosal Lesions, and MPO Activity

**Figure 3:**
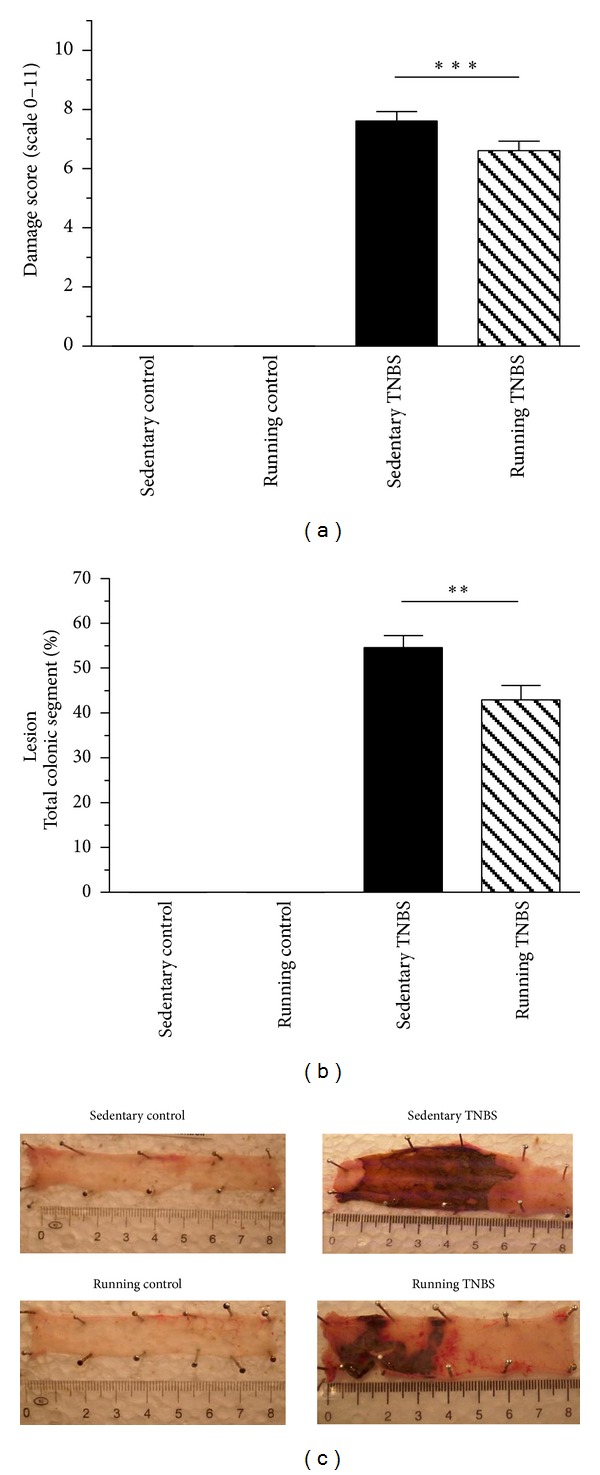
Effects of 6 weeks of running on macroscopic colonic inflammatory damage score (a) and lesion (b) in TNBS-induced colitis after 72 hrs. Results are shown as mean ± S.E.M. (**P < 0.01 and ***P < 0.001 compared to the sedentary TNBS group, *n* = 13–19). Representative images of freewheel running and induction of colonic inflammation: sedentary control, and running control, sedentary TNBS, running TNBS (c).

The severity of mucosal damage was scored on a 0–11 scale by a randomized, blinded fashion. 6 weeks of running before the TNBS challenge significantly (*P* < 0.001) decreased the level of this inflammatory damage score marker from 7.6 ± 0.3 to 6.6 ± 0.3 (Figure 3(a)).

Challenge with TNBS caused hemorrhagic and necrotic damage of the colonic mucosa, as determined macroscopically 72 hours after TNBS administration. In the sedentary, nonrunning group the level of damage reached 54.6 ± 2.63% of the total colonic area. We detected a significant decrease (42.9 ± 3.21%, *P* < 0.01) in macroscopic injury in the colons of the trained, TNBS-treated group (Figures 3(b) and 3(c)).

**Figure 4:**
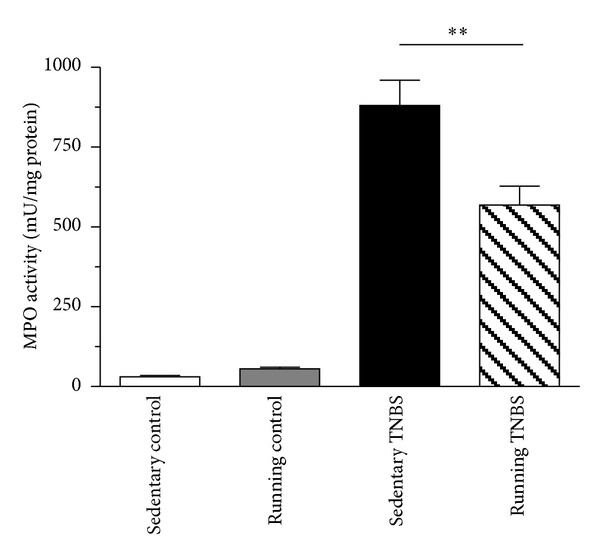
MPO activity in rats treated with TNBS after 6 weeks of resting or 6 weeks of running. Results are shown as mean ± S.E.M. (**P < 0.01 compared to the sedentary TNBS group, *n* = 7–16).

Following treatment with TNBS, there was a thirtyfold increase in colonic MPO activity, suggesting massive neutrophil infiltration [22]. Wheel running significantly (*P* < 0.01) attenuated the elevation of MPO activity from 880.6 ± 79.3 to 568.4 ± 59.9 mU/mg protein (Figure 4).

### 3.3. The Effect of Physical Exercise on the Gene Expression Profiles of Selected Pro- and Anti-Inflammatory Mediators in TNBS-Induced Experimental Acute Colitis

**Figure 5:**
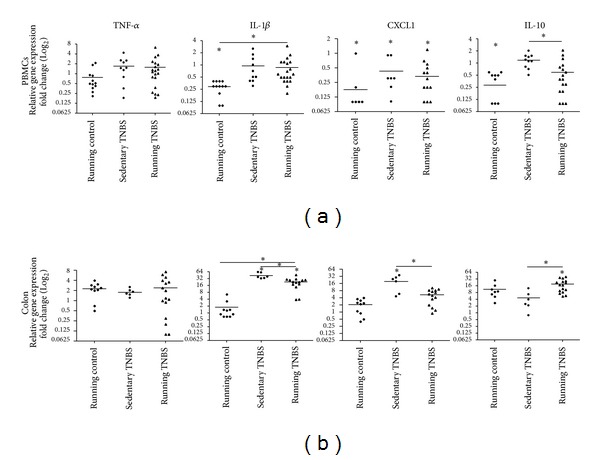
The relative gene expression of selected pro- and anti-inflammatory mediators in peripheral blood mononuclear cell (PBMC) (a) and colon (b). The expression pattern of TNF-*α*, IL-1*β*, CXCL1, and IL-10 from sedentary and running groups with or without TNBS treatment was determined with qPCR and compared to sedentary nontreated animals (absolute controls). Results are shown as data points and median (*P < 0.05; *n* = 6–20).

In PBMCs, the physical exercise alone significantly (*P* < 0.05) downregulated the gene expression of IL-1*β*, CXCL1 and IL-10 as compared to the inactive group (Figure 5(a)). TNBS treatment alone only downregulated the expression of CXCL1, and had no effect on the expression of TNF-*α*, IL-1*β*, and IL-10 transcripts. Interestingly, exercise before TNBS treatment downregulated the expression of CXCL1 and IL-10. Notably, the downregulation of IL-10 was significant as compared to not only absolute controls but also to TNBS treatment alone (Figure 5(a)).

In contrast to the downregulated expression pattern detected in PBMCs, physical exercise alone did not alter the gene expression of the monitored inflammatory mediators in the colon when compared to sedentary controls (Figure 5(b)). Yet, TNBS treatment alone induced marked upregulation (*P* < 0.05) in the expression of pro- but not anti-inflammatory mediators (IL-1*β*, CXCL1, and IL-10, resp.) in the colon. Furthermore, exercise significantly attenuated TNBS-induced upregulation of proinflammatory mediators (IL-1*β*, CXCL1) and significantly increased expression of anti-inflammatory IL-10 (Figure 5(b)).

### 3.4. Effect of Running and/or TNBS Challenge on Colonic TNF-*α* Protein Content

**Figure 6:**
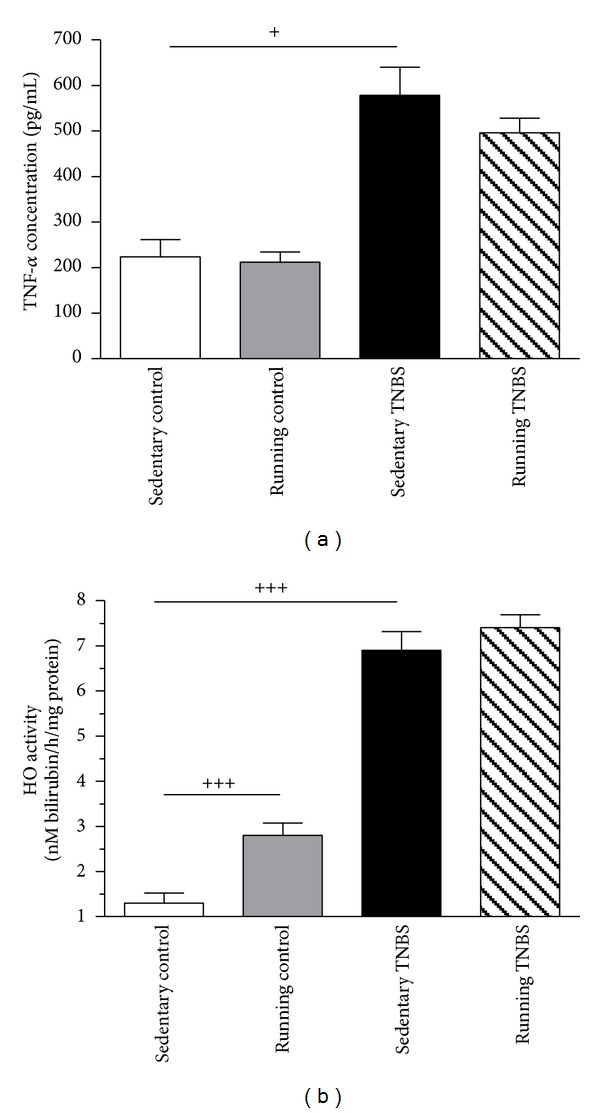
(a) Concentration of TNF-*α* in 1 g tissue in sedentary and running groups with or without TNBS treatment. Results are shown as mean ± S.D. (^+^ *P* < 0.05; *n* = 4–10). (b) Effects of 6 weeks of running on colonic HO activity either in the control group or in TNBS-induced colitis after 72 hrs. Results are shown as mean ± S.E.M. (^+++^ *P* < 0.001 compared to the sedentary nontreated control group, *n* = 8–16).

6-week running had no significant effect on the colonic content of TNF-*α*. In the inflamed colons we found a doubling of TNF-*α* concentration in the sedentary group (578.4 ± 118.47 pg/mL), and although 6-week running before TNBS treatment slightly decreased TNF-*α* levels (496.16 ± 87.77 pg/mL), the decrease was not significant compared to the sedentary TNBS group (Figure 6(a)).

### 3.5. Changes in Colonic HO Activity by Physical Exercise and/or TNBS Treatment

Voluntary physical exercise significantly increased HO activity from 1.3 ± 0.2 to 2.8 ± 0.3 nmol bilirubin/h/mg protein (*P* < 0.001). Treatment with TNBS alone led to even higher HO activity (6.9 ± 0.4 nmol bilirubin/h/mg protein, *P* < 0.001), after 6 weeks of exercise slightly increased the HO activity, but this difference was not significant between the running TNBS group and the nonrunning TNBS group (Figure 6(b)).

### 3.6. Effects of TNBS Treatment and/or Physical Exercise on Colonic cNOS Activity

**Figure 7:**
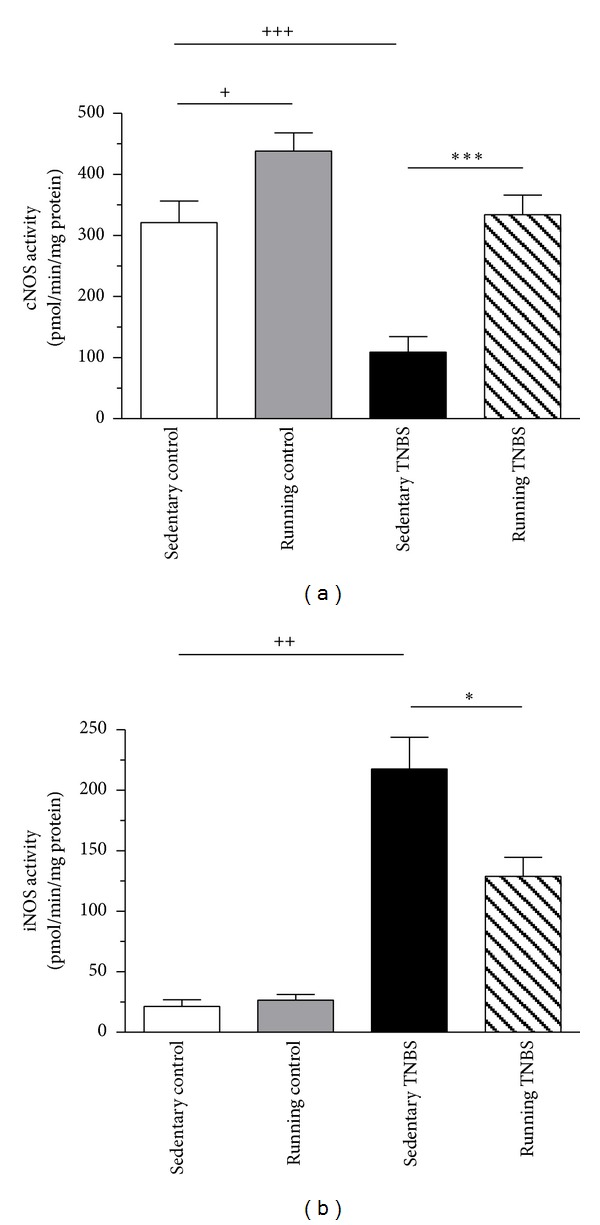
Effects of 6 weeks of running on colonic cNOS activity (a) and iNOS activity (b) either in the control group or in TNBS-induced colitis after 72 hrs. Results are shown as mean ± S.E.M. (^+^ *P* < 0.05, ^++^ *P* < 0.01, and ^+++^ *P* < 0.001 compared to the sedentary control group and *P < 0.05, ***P < 0.001 compared to the sedentary TNBS group, *n* = 6–15).

The 6-week running alone caused a significant (*P* < 0.05) increase in cNOS activity. In the nonrunning TNBS group we have measured a significantly decreased (*P* < 0.001) cNOS activity (from 321.1 ± 35.16 to 108.9 ± 25.6 pmol/min/mg protein) compared to the nontreated control group. Voluntary exercise before TNBS administration significantly (*P* < 0.001) augmented cNOS activity from 108.9 ± 25.6 to 333.9 ± 32.3 pmol/min/mg protein compared to the nonrunning TNBS group (Figure 7(a)).

### 3.7. Effects of TNBS Treatment and/or Physical Exercise on Colonic iNOS Activity

Inflammation caused by TNBS challenge significantly elevated (*P* < 0.01) iNOS activity (from 21.4 ± 5.49 to 217.5 ± 26.43 pmol/min/mg protein) independently of exercise. Importantly, the 6-week running before TNBS treatment significantly (*P* < 0.05) reduced this increase of iNOS activity from 217.5 ± 26.43 to 128.9 ± 15.82 pmol/min/mg protein (Figure 7(b)).

## 4. Discussion

The results of the present study demonstrate that 6-week physical exercise before TNBS challenge can ameliorate the severity and extent of colonic damage caused by TNBS treatment in rats by downregulating the expression of proinflammatory mediators, inducing the expression of anti-inflammatory mediators, and affecting the activity of cNOS and iNOS enzymes.

In this study we used the 2,4,6-trinitrobenzene-sulfonic-acid- (TNBS-) induced rat acute colitis model, in which the inflammatory response is due to the generation of transmural oxidative stress and release of proinflammatory mediators [26, 27]. We used a voluntary wheel running model to mimic leisure-type physical exercise, which induces lower degree of stress compared to swimming or treadmill exercise protocols due to the reduced handling of animals and stress-inducing exercise motivators [28].

The body weights after 6 weeks of running did not show any differences compared to the nonrunning groups, however, body composition was not examined.

Intracolonic administration of TNBS causes an acute, severe, transmural inflammation, which mimics several clinical and morphological features of Crohn's disease [26]. In the sedentary colitis group on average, 50% of the 8 cm colonic portion was inflamed with ulceration and we have also found a 30-fold elevation in MPO activity.

In order to determine if gene expression changes of PBMCs reflect that of the inflamed colon, we have monitored the gene expression changes of well-known inflammatory markers. Interestingly, we found that 6 weeks of running alone downregulated the expression of both pro- and anti-inflammatory mediators (IL-1*β*, CXCL1, and IL-10, resp.) in PMBCs but not in the colon. In addition, in the colon physical exercise only attenuated the expression level of anti-inflammatory IL-10 compared to sedentary TNBS-treated animals.

In contrast to PBMCs, TNBS induced marked upregulation of proinflammatory cytokines IL-1*β* and CXCL1 in the colon, which is in good agreement with previous studies showing elevated expression of these molecules in IBD [6]. Importantly, recreational physical exercise downregulated TNBS-induced expression of both cytokines. In parallel, we detected markedly elevated IL-10 expression in running TNBS animals. These observations, together with those showing decreased level of damage score in physically active animals, suggest that physical activity indeed ameliorates the extent of the inflammation caused by TNBS. However, it remains to be determined if physical activity shows a similar protective effect in patients with chronic IBD.

Although there was no change in TNF-*α* gene expression we found a significantly increased TNF-*α* protein content in the inflamed colon with TNBS in accordance with earlier findings [26, 29]. We hypothesize that polymorphism in TNF-**α** promoter regions could be responsible for discrepancies between gene and protein expression level [30]. However, no significant decrease in TNF-*α* protein content was seen after exercise in the TNBS-treated group compared to the nonrunning TNBS-treated group. It is feasible that exercise impacts inflammation through pathways other than TNF-*α*, because several other markers were attenuated in our study, indicating the effectiveness of this intervention against IBD.

The elevated HO activity suggests the involvement of oxidative stress in colonic damage. Earlier findings demonstrated the ability of chemically induced HO to reduce the extent and intensity of experimental colonic inflammation [14]. Several studies demonstrated that long-lasting exercise can induce antioxidant mechanisms in a variety of tissues, probably as a defensive response to oxidative stress [31]. Brooks et al. [32] demonstrated that 8 weeks of treadmill running reduced the release of ROS and NO from contracting muscles and increased skeletal muscle antioxidant content such as glutathione and protein thiols in mice. George et al. [33] found an elevated activity and expression of superoxide dismutase and HO-1 in the plasma and renal proximal tubules of rats after treadmill training for 12 weeks. Our observations that a 6-week voluntary exercise can induce colonic HO activity also agree with previous data, supporting the antioxidant effects of moderate training. Along these lines, Kasimay et al. demonstrated the protective role of 6 weeks of wheel running against oxidative colonic injury in a model of acetic acidinduced intestinal inflammation [34].

We found opposite changes of cNOS and iNOS activity in the inflamed colon. Our observations of decreased cNOS and increased iNOS activity in the sedentary colitis group are also consistent with previous findings. We do not know to what extent the two constitutive isoforms (nNOS and eNOS) are responsible for the reduction of cNOS activity. Experiments with eNOS deficient mice show the important role of the other constitutive NOS isoform in limiting intestinal injury in IBD [35]. In IBD nNOS is downregulated, while iNOS expression increases cyclically during the active phase of inflammation. Excess NO produced by iNOS can generate the prooxidant peroxynitrite [36]. This overproduction of NO by upregulation of iNOS seems to be responsible for the observed intestinal hypomotility and subsequent bacterial overgrowth [18].

We presented that rat colonic cNOS activity increased, while iNOS activity remained unchanged with exercise compared to the sedentary control group. Together with the observed expression changes induced by TNBS, it appears that prior exercise is able to mitigate colonic inflammation and macroscopic damage by maintaining cNOS and attenuating iNOS expression in IBD. These observations are also in agreement with previous data, showing that regular physical activity induces increased eNOS gene expression and reduces the expression of iNOS in CD34(+) PBMCs [37]. Moreover, NO-releasing derivative of aspirin can accelerate the healing of colonic inflammation in an animal model [38]. Our results are in agreement with earlier studies about naturally occurring compounds and drugs such as naringenin, a flavonoid in citrus, grapefruits, and tomatoes [39] 3,4-oxo-isopropylidene-shikimic acid [40] which has anti-inflammatory effect on experimental colitis through reduction of the expression of inflammatory cytokines and mediators such as iNOS. Therefore, it appears that the NO pathway plays a central role in the resolution of inflammation in IBD.

Exercise has been speculated to be protective against the onset of IBD, but the literature is inconsistent. Preliminary studies reveal that exercise training (relatively low intensity) may be beneficial to reduce stress and symptoms of IBD. Current research also recommends exercise to help counteract some IBD-specific complications by improving bone mineral density, immunological response, psychological health, weight loss, and stress management ability. However, the literature advises that some patients with IBD may have limitations to the amount and intensity of exercise that they can perform [2].

It has become clear that physical activity is not harmful for patients with inflammatory bowel disease, despite acute exercise related responses, such as increased serum malondialdehyde levels and activated neutrophils. In addition, physical activity may reduce disease activity and improve physical health, general well-being, and perceived stress. In summary, exercise may be beneficial to IBD patients, but further research is required to make a convincing conclusion regarding its role in the management of IBD and to help establish exercise regimens that can account for each IBD patient's unique presentation [2]. In conclusion, in the present study we aimed to investigate young adult rats, which are age-matched population to the incidence of IBD [41]. Our results suggest that exercise may be protective against the onset of IBD through the modulation of pro- and anti-inflammatory and antioxidant mediators with HO and NOS enzymes playing a central role in these effects. Along these lines, regular exercise can promote healthy aging via the same mechanisms.
